# Pain and neuropsychiatric symptoms in community-dwelling people living with dementia: a cross-sectional dyadic study

**DOI:** 10.1186/s12877-026-07755-6

**Published:** 2026-06-06

**Authors:** Jemima T. Collins, Alison Cowley, Tom Dening, Claudio Di Lorito, Tristan Emerson, Catherine Fullwood, Rowan H. Harwood, Denise Kendrick, Daniel F. McWilliams, Amanda Roberts, Gurvinder Sahota, John Gladman

**Affiliations:** 1 Injury, Recovery and Inflammation Sciences, School of Medicine, University of Nottingham, Nottingham, NG7 2RD UK; 2 University Hospitals of Derby and Burton NHS Foundation Trust, Derby, UK; 3 Research & Innovation, Nottingham University Hospitals NHS Trust, Nottingham, UK; 4 NIHR East Midlands RRDN, University Hospitals of Leicester NHS Trust, Leicester, UK; 5 School of Medicine, Mental Health & Clinical Neurosciences, Nottingham, UK; 6 Primary Care and Population Health, University College London, London, UK; 7 Research & Innovation, Centre for Biostatistics, Division of Population Health, Manchester University NHS Foundation Trust, University of Manchester, Manchester, UK; 8 School of Health Sciences, University of Nottingham, Nottingham, NG7 2HA UK; 9 Centre for Academic Primary Care, Lifespan and Population Health, School of Medicine, University of Nottingham, Nottingham, NG7 2RD UK; 10 Arthritis-UK Pain Centre, School of Medicine, NIHR Nottingham BRC, IRIS (Injury, Recovery and Inflammation Sciences), Nottingham, UK

**Keywords:** Pain, Dementia, Neuropsychiatric symptoms

## Abstract

**Background:**

Pain may be linked to neuropsychiatric symptoms in dementia but the nature of these associations is unclear. The aims of this study were to examine the concurrent validity of pain scales and to investigate the relationship between pain and neuropsychiatric symptoms in community-dwelling people with dementia.

**Methods:**

This study recruited dyads of people with dementia and their caregivers. Questionnaires measuring pain (Numeric Rating Scale, Brief Pain Inventory Short Form, EQ5D3L) and cognition (Mini- Addenbrookes Cognition Examination) were completed by people with dementia. Dyad partners completed the Neuropsychiatric Inventory Questionnaire (NPI-Q). We examined the degree of agreement between different measures of pain and compared the NPI-Q score and items between people with dementia with and without troublesome pain (NRS ≥ 4, NRS < 4), and between those with single and multisite pain.

**Results:**

The study recruited 35 individuals with dementia (49% female, mean age 80 with a range of 65–91 years, mean Mini-ACE 13.6/30) and 35 dyad partners. 66% of people with dementia had troublesome pain and compared to those without troublesome pain they were not significantly different in terms of age, sex, frailty, or other demographic variables, but were more likely to take regular paracetamol.

Agreement between the NRS and EQ5D3L pain score was good (Cohen’s kappa 0.70, *p*<0.001), as was agreement between the NRS and BPI-SF with a close to 0 mean difference between the scores on a Bland-Altman plot. The total NPI-Q score was not significantly associated with the presence of troublesome pain. Of all NPI-Q symptoms, only disinhibition showed associations with both painseverity and multisite pain (44% vs 0% and 38% vs 0% respectively, both *p*<0.05).

**Conclusion:**

Community-dwelling people living with dementia can self-report pain using a range of pain scales.

Certain neuropsychiatric symptoms may be more common in the presence of pain but requires further study with larger cohorts.

## Background

Dementia is a global health priority, with a forecasted 152.8 million people living with dementia by the year 2050 [1]. In the absence of a cure, the emphasis of care is on improving symptoms, promoting independence and optimising quality of life [2]. Pain is highly prevalent in people with dementia (50–70% in community-dwelling people with. dementia) [3] and causes distress, reduced quality of life and impaired functional independence in people who already struggle with the impacts of dementia [4]. Dementia interacts with pain by reducing normal pain inhibitory responses, and communication impairments contribute to under-recognition of pain [5, 6]. This leads to pain being under-recognised and sub-optimally managed and is a key unmet need which may result in manifestation of behavioural or neuropsychiatric symptoms of dementia [7].

Almost everyone with dementia displays one or more neuropsychiatric symptom across the course of their illness [8]. Neuropsychiatric symptoms in dementia are caused by multiple factors such as the severity of cognitive impairment and unmet needs [7]. The relationship between pain and neuropsychiatric symptoms in people with dementia may be bidirectional: a systematic review of interventions for both pain and neuropsychiatric symptoms found that interventions aimed at improving pain reduced neuropsychiatric symptoms, and interventions targeting neuropsychiatric symptoms tended to reduce pain [9].

For people with dementia living in care homes, the neuropsychiatric symptoms with the strongest associations with pain are depression, agitation and aggression [10]. These associations may not be present in community-dwelling people with dementia because they may have less severe cognitive impairment as well as markedly different physical and social environments. Given the potentialbidirectional relationship between pain and neuropsychiatric symptoms, identification of specific pain-related neuropsychiatric symptoms could justify non-pharmacological or pharmacological management approaches for pain or neuropsychiatric symptoms to ameliorate both.

Commonly used self-reported pain scales such as the Numeric Rating Scale have poorly described usability and validity for people with dementia [11]. Proxy, objective pain scales, such as the PAINAD scale [12], utilises observation of changes in breathing, vocalisation, consolability and facial expression, and was produced specifically for those with advanced dementia. There exists equipoise for the most appropriate scale, in people with mild to moderate dementia whose ability to self-report may be impaired, yet retain the ability to communicate their needs. Self-reported tools are considered the most reliable indicator of pain, and the choice of pain scale in dementia should be based on the person’s ability to use the scale. Reliability of responses is enhanced by additional time given to complete these scales and effective communication and prompts by the assessor [13].

Establishing whether people with mild dementia can use common self-reported pain scales such as the Numeric Rating Scale (NRS), Brief Pain Inventory-Short Form (BPI-SF) and the pain domain in Euroqol 5D-3 L (EQ5D3L) [14–16] to report pain is unclear yet important both for clinical practice and research.

### Aims

The aims of this study were to examine the agreement (concurrent validity) of self-reported pain measures and to investigate the relationship between pain and neuropsychiatric symptoms in community-dwelling people with dementia.

## Methods

### Study design and procedure

This study was a cross-sectional, descriptive study using validated health status questionnaires. Recruitment was from the East Midlands in the United Kingdom, spanning counties of Nottinghamshire, Derbyshire and Leicestershire, through five routes of identification: primary care practices, one National Health Service elderly care outpatient department, a community Memory Assessment Service, the National Institute of Health and Care Research (NIHR) Join Dementia.

Research (JDR) database, and public engagement events. For primary care practices, hospital outpatient departments and the Memory Assessment Service, potentially eligible participants identified by the direct care team were signposted to the research team, and given a Participant Information Sheet to consider the study further. JDR is a platform where people living with dementia interested in participating in research can sign up, consenting to being contacted by research teams if they match recruitment criteria. Members of the research team also actively engaged with individual people living with dementia, their caregivers and over 20 community groups where people with dementia meet, leading to invitations to speak at 15 face-to-face community events to draw attention to the study. Potential participants who expressed an interest to take part in the study were screened against inclusion and exclusion criteria, and if eligible, an interview date was then agreed. 

Data were collected by trained researchers through face-to-face interviews in participants’ homes or university sites, according to participant preference. Ethical approval was granted by the Greater Manchester South Research Ethics Committee, REC reference 24/NW/0027.

### Participants

Individuals over 65 years of age living in the community with self- or consultee-reported mild to moderate Alzheimer’s, vascular or mixed dementia were invited to participate. People with dementia and chronic pain, as defined by persistent pain lasting 3 months or more and people with dementia with no chronic pain were included to enable comparisons between groups with pain and without pain. Participants were recruited as dyads, which were pairs comprising a person living with dementia and a family member, close friend or caregiver, who was referred to as the dyad partner. 

The only exclusion criteria for the dyad partner was < 18 years of age and presence of cognitive impairment.

All researchers conducting face-to-face interviews with the participants were registered healthcare professionals and were trained to assess participants’ mental capacity to consent to a research study. The person with dementia gave informed consent if they had capacity to do so. If they did not have the capacity to do so, agreement to participate in the study was given by their consultee, who in most cases was the dyad partner. Dyad partners gave their own consent to participate. A dyadic recruitment approach was chosen in order that the dyad partner could support the person with dementia in their participation in the study, assist with completion of the questionnaires where required, and report neuropsychiatric symptoms in the neuropsychiatric inventory questionnaire (NPI-Q) [17].

### Study measures

Demographic variables of age, sex, ethnicity and Index of Multiple Deprivation (which scores neighbourhood areas for socioeconomic deprivation [18]) were captured. The researcher also assessed dependency for activities of daily living (ADL) and frailty using the Katz ADL score (0 being very dependent and 6 being independent) [19] and the Clinical Frailty Scale (1 being fit and 9 being terminally ill) respectively [20], by assessing the participant with dementia directly. 

Severity of pain was self-reported using the NRS, BPI-SF and the pain domain from the EQ5D3L scale [14–16]. The NRS was used as the reference standard self-reported pain scale [21]. NRS and BPI-SF ascertained pain severity on a scale of 0–10 where 0 corresponds to no pain and 10 worst possible pain. NRS was a single question ‘In the past week, how bad was your pain (where 0 is ‘no pain’, and 10 is ‘pain as bad as could be’)?’. To allow comparison of agreement with the NRS, the BPI-SF question ‘Please rate your pain by marking the box beside the number that best describes your pain at its worst in the last 24 hours’ was used. To allow comparison of agreement with the NRS, the EQ5D3L Pain responses used were: ‘I have no pain or discomfort’, ‘I have moderate pain or discomfort’ and ‘I have extreme pain or discomfort’. BPI-SF ascertained pain distribution using a pain manikin. Neuropsychiatric symptoms were reported by dyad partners using the Neuropsychiatric Inventory Questionnaire (NPI-Q) which included the number and severity of neuropsychiatric symptoms present, as well as level of caregiver distress [17]. The Mini-Addenbrookes Cognitive.

Examination (Mini-ACE), conducted by the researcher, is a valid and reliable measure of global cognition in people with dementia [22]. All these measures were completed by the participant with dementia, apart from the NPI-Q which is designed as an informant questionnaire.

### Analysis

Data were presented descriptively subdivided according to levels of pain – troublesome pain (NRS ≥ 4) with a patient-rated threshold of 4 corresponding to unacceptable pain and interference with functioning, and no troublesome pain (NRS < 4) [14, 23]. The agreement between measures of self-reported pain was assessed using Bland-Altman plots for continuous data and Cohen’s kappa for categorical data. Associations between pain and outcomes were explored using cross-tabulation, Fisher’s exact and Mann-Whitney U tests where appropriate and presented using odds ratios and 95% confidence intervals.

## Results

A total of 35 people with dementia (PwD) and 35 of their dyad partners were recruited. PwD were 49% female with a mean (SD) of age of 79.6 (6.5) years, and age range of 65–91 years. Dyad partners were 69% female with a mean (SD) age of 72.5 (10.7) years, 29/35 (83%) lived with the PwD, whilst the remaining 17% saw the PwD one or more times a week. Twenty-three (66%) PwD had pain that was considered troublesome (NRS ≥ 4) and they were compared to the 12 (34%) with less troublesome pain (NRS < 4).

PwD with pain were not significantly different from those with no pain in terms of age, sex, frailty, deprivation levels, cognition or dependence on others for ADLs. PwD with pain were more likely to take regular paracetamol compared to those without pain (91% vs. 58%, *p* = 0.014) (Table 1).

**Table 1:** Description of troublesome pain vs. no pain

	Troublesome Pain Group, NRS ≥ 4 (*n* = 23)	Less Pain Group, NRS < 4 (*n* = 12)	*p*-value
Sex, *n* (%)			
Male	11 (48)	7 (58)	0.73
Female	12 (52)	5 (42)	
Age range, *n* (%)			
65–74	5 (22)	2 (17)	0.71
75–84	12 (52)	8 (66)	
85+	6 (26)	2 (17)	
Ethnicity, *n* (%)	22 (96)	12 (100)	1.0
White ethnicity	1 (4)	0 (0)	
Non white ethnicity, *n* (%)			
Index of multiple deprivation (IMD), *n* (%)			
Quintile 1 (most deprived)	1 (4)	0 (0)	0.97
Quintile 2	1 (4)	1 (8)	
Quintile 3	6 (26)	2 (17)	
Quintile 4	5 (22)	3 (25)	
Quintile 5 (least deprived)	10 (43)	6 (50)	
Lives with, *n* (%)			
Alone	3 (13)	2 (17)	0.68
Spouse/Partner	17 (74)	10 (83)	
Other family members	3 (13)	0 (0)	
Clinical Frailty Scale, *n* (%)			
2 (well)	1 (4)	2 (17)	0.44
3 (managing well)	2 (9)	1 (8)	
4 (vulnerable)	11(48)	5 (42)	
5 (mildly frail)	5 (22)	4 (33)	
6 (moderately frail)	4 (17)	0 (0)	
Mini ACE, *n* (%)			
< 21 (poorer cognition)	16 (70)	11 (92)	0.22
≥ 21 (better cognition)	7 (30)	1 (8)	
Katz Index of Independence of ADL, *n* (%)			
Independent for all ADLs	9 (39)	8 (67)	0.16
Dependent in ≥ 1 ADL category	14 (61)	4 (33)	
Pain Medication, *n* (%)			
Paracetamol*	[1]		
Yes	21 (91)	7 (58)	**0.01**
No	1 (4)	5 (42)	
Weak or strong opioids*	[1]		
Yes	11 (48)	2 (17)	0.08
No	11 (48)	10 (83)	
Neuropathic agents*	[1]		
Yes	7 (30)	1 (8)	0.21
No	15 (65)	11 (92)	
Other analgesia (over the counter, steroid injections, NSAIDs oral or gel)	[1]		
Yes	7 (30)	3 (25)	1.00
No	15 (65)	9 (75)	
NPI-Q total scores by domain			
Total number of NPI-Q behaviours, median (IQR)	6.00 (3.00–8.00)	4.00 (3.00–6.00)	0.21^
Total severity of NPI-Q behaviours, median (IQR)	7.00 (5.00–15.00)	7.50 (4.25–9.50)	0.50^
Total distress by NPI-Q behaviours, median (IQR)	11.00 (7.00–23.00)	12.00 (4.00–15.00)	0.44^

[ ] Number of cases of missing values for this particular analysis

Difference between groups tested using the Fisher’s exact test in all except for those indicated by ^which were analysed using the Mann Whitney U test. Results in bold are statistically significant

*Mini ACE *Mini Addenbrooke’s Cognitive Assessment, *ADL *Activities of Daily Living, *NSAIDs *Non-steroidal anti-inflammatory drugs, *NPI-Q *neuropsychiatric inventory questionnaire

**Fig. 1:**
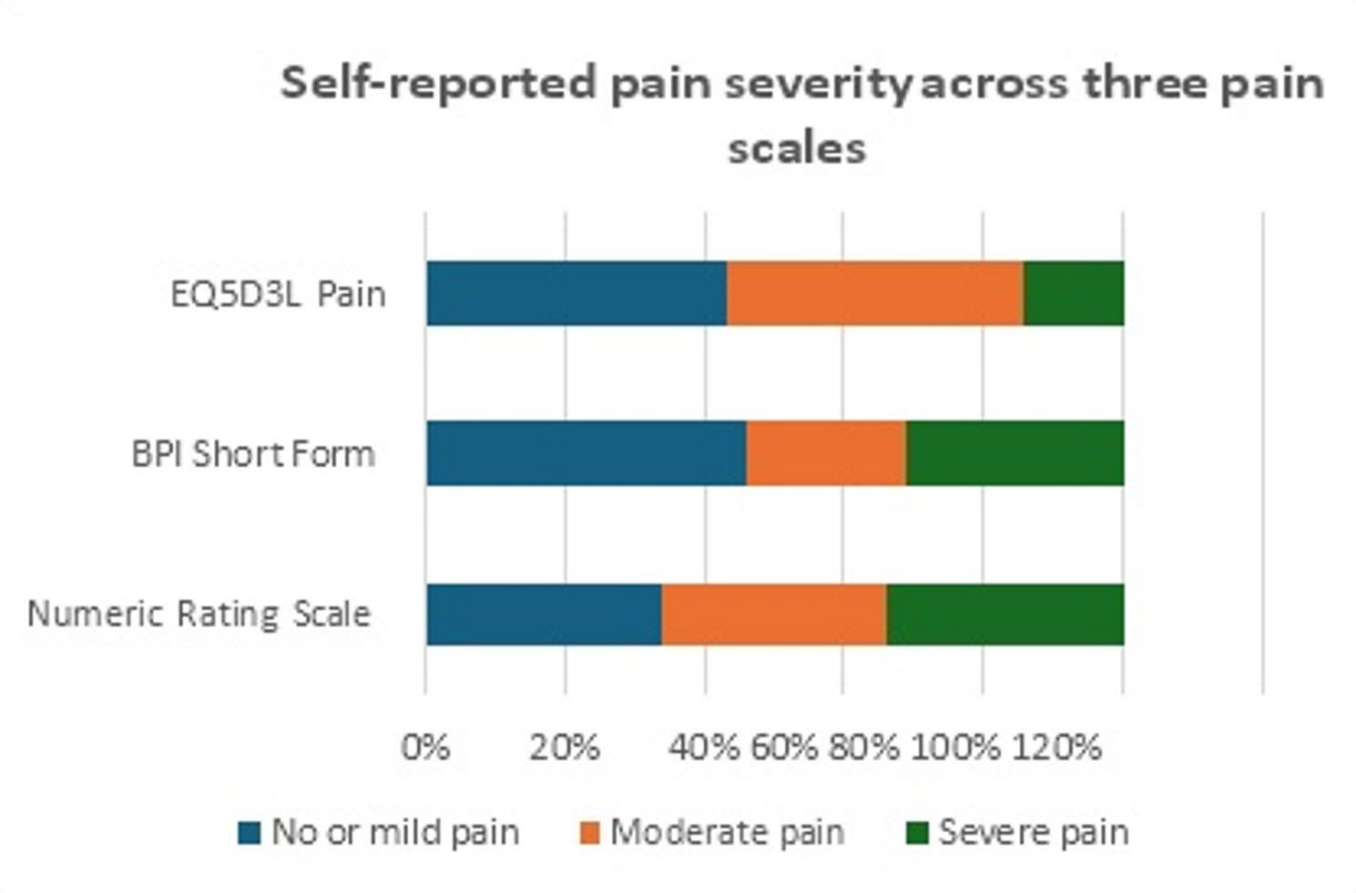
Bar chart of percentages of people reporting different pain severity using three different pain scales

Self-reported pain severity was presented graphically (Fig. 1) across three scales, categorising into no or mild pain (NRS and BPI 0–3), moderate pain (NRS and BPI 4–6) and severe pain (NRS and BPI 7–10) [24].

**Fig. 2:**
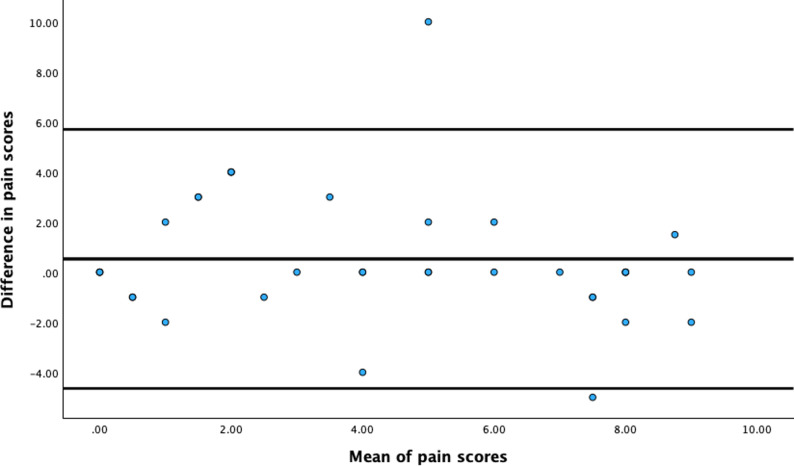
Agreement between self-reported pain using the Numeric Rating Scale (NRS) and worst pain in the Brief Pain Inventory Short Form scales

Agreement between the NRS and BPI-SF pain scales was explored with a Bland-Altman plot. The Bland-Altman plot showed that the mean difference between the scores (0.53) was close to 0 with 33 out of 35 data points scattered relatively closely around this mean, and only two data points outside of the 95% CI (Fig. 2). No specific trend was observed in agreement at lower or higher mean pain values. Overall, this suggests good agreement in the reporting of pain using these scales (Fig. 2).

Agreement between NRS and EQ5D3L pain domain scores was assessed using Cohen’s Kappa. Equation 5D3L pain was dichotomised into two levels of no pain and pain (moderate or extreme pain or discomfort). The NRS was dichotomised into two levels of no troublesome pain (NRS < 4) and troublesome pain (NRS ≥ 4) [14]. Comparing both these scores showed good agreement (0.70) (Table 2).

**Table 2:** Agreement of self-reported pain for people living with dementia, according to the Numeric Rating Scale (NRS) and Euroqol 5D3L (EQ5D3L) scales

	NRS Pain ≥ 4, *n* (%)	NRS Pain < 4, *n* (%)
EQ5D3L Moderate or Severe Pain *n*	19 (83)	1 (8)
EQ5D3L No pain, n	4 (17)	11 (92)

Cohen’s kappa 0.701, *p* < 0.001

There were no statistically significant differences in the collective number of neuropsychiatric symptoms, the severity of the symptoms or caregiver distress from the symptoms comparing people with troublesome pain and no pain (Table 1).

Neuropsychiatric symptoms were reported by the dyad partner, and apathy was the most common (23/35) and euphoria/elation the least (6/35). The associations between pain and neuropsychiatric symptoms are presented in Table 3. Disinhibition and apathy had statistically significant associations with pain (Table 3). People with two or more painful sites were less likely to report experiencing apathy compared to people with no or single site pain (OR 0.65) (Table 3). Disinhibition was present in 10/23 (44%) of people with greater pain severity (vs. 0% in people with less pain) and in 10/26 (38%) of people with 2 or more sites of pain (vs. 0% in people with no pain or single site pain), both p < 0.05) (Table 3).

**Table 3:** Relationship between NPI-Q items and troublesome /non-troublesome pain and multisite/single site pain

Pain						
Presence of individual NPI-Q domains	Troublesome Pain Group, NRS ≥ 4, *n* (%)	Less Pain Group, NRS < 4, *n* (%)	Odds ratio (95% CI)	Pain ≥ 2 sites, *n* (%)	Single site or no pain, *n* (%)	Odds ratio (95% CI)
Delusions						
Yes	10 (44)	3 (25)	1.74 (0.59,	11 (42)	2 (22)	1.90
No	13 (56)	9 (75)	5.15)	15 (58)	7 (78)	(0.52, 7.00)
Hallucinations						
Yes	11 (48)	2 (17)	2.87 (0.76,	12 (46)	1 (13)	4.15
No	12 (52)	10 (83)	10.91)	14 (54)	8 (87)	(0.63, 27.60)
Agitation/Aggression						
Yes	14 (61)	5 (42)	1.46 (0.69,	17 (65)	2 (22)	2.94
No	9 (39)	7 (58)	3.08)	9 (35)	7 (78)	(0.84, 10.31)
Dysphoria/Depression						
Yes	17 (74)	5 (42)	1.77 (0.87,	20 (77)	2 (22)	3.46
No	6 (26)	7 (58)	3.62)	6 (23)	7 (78)	(1.00, 11.97)
Anxiety						
Yes	15 (65)	7 (58)	1.12 (0.64,	18 (69)	4 (44)	1.56
No	8 (35)	5 (42)	1.96)	8 (31)	5 (56)	(0.72, 3.38)
Euphoria/Elation*						
Yes	6 (26)	0 (0)	p-value=	5 (19)	1 (13)	1.73
No	17 (74)	12 (100)	0.062	21 (81)	8 (87)	(0.23, 12.90)
Apathy/Indifference						
Yes	14 (61)	9 (75)	0.81 (0.51,	15 (58)	8 (87)	**0.65**
No	9 (39)	3 (25)	1.29)	11 (42)	1 (13)	**(0.43**, **0.97)**
Disinhibition*						
Yes	10 (44)	0 (0)	**p-value=**	10 (38)	0 (0)	**p-value=**
No	13 (56)	12 (100)	**0.006**	16 (62)	9 (100)	**0.029**
Irritability/Lability						
Yes	13 (57)	8 (67)	0.85 (0.50,	18 (69)	3 (33)	2.08
No	10 (43)	4 (33)	1.45)	8 (31)	6 (67)	(0.80, 5.42)
Motor Disturbance						
Yes	6 (26)	3 (25)	1.04 (0.32,	7 (27)	2 (22)	1.21
No	17 (74)	9 (75)	3.46)	19 (73)	7 (78)	(0.31, 4.80)
Nighttime disturbance						
Yes	11 (48)	5 (42)	1.15 (0.52,	13 (50)	3 (33)	1.50
No	12 (52)	7 (58)	2.54)	13 (50)	6 (67)	(0.55, 4.08)
Appetite/Eating						
Disturbances						
Yes	12 (52)	5 (42)	1.25 (0.58,	14 (54)	3 (33)	1.62
No	11 (48)	7 (58)	2.72)	12 (46)	6 (66)	(0.60, 4.35)

OR (95% CI) are not reported where cells contain zero, Elation/Euphoria* and Disinhibition*, here the p-value obtained using Fisher’s Exact test is reported

Results in bold are statistically significant

## Discussion

This study described and characterised the measurement of pain and its association with neuropsychiatric features in community-dwelling people with non-advanced dementia. In this cohort of people with dementia, we found that there was agreement in measures of self-reported pain. We also found people with troublesome or multisite pain were more likely to display disinhibition, and less likely to report apathy.

Self-reported pain scales were developed for anyone with the ability to self-report pain. In people with dementia, pain scales are often designed for use by an observer and include non-verbal prompts and behaviour observation [11], specific to those with advanced dementia. If a person with less advanced dementia has the ability to self-report pain, they should be given the opportunity to do so. If a person has communication challenges and cognitive impairment that prevents self-assessment of pain, then the next best option is an assessment from a person with high familiarity with them [25]. We found that self-reported pain scales used in this population had good agreement (concurrent validity), and were feasible for completion either by the person with dementia themselves or supported by their dyad partner. This indicates that these pain scales can be used in this population and may also be used clinically to characterise pain, with or without the support of a partner, compared with informal clinical questioning alone.

Out of all the neuropsychiatric symptoms, only disinhibition was significantly more likely to occur in the presence of troublesome pain and multisite pain, compared to no pain and single site or no pain respectively. Disinhibition in dementia is characterised by behaving impulsively, inappropriately, or saying things which are rude or hurtful. Although this could have been a chance finding, disinhibition may be a useful marker to prompt enquiry about pain management. Apathy was less likely to occur in the presence of multisite pain, compared to no pain and single site pain. People with a lack of apathy may have more pain as they are more active, compared to those with apathy. Together with the significant association with disinhibition, these neuropsychiatric symptom profiles are markers of pain severity and distribution, and may be prompts for clinical action.

This study adds to existing literature in the characterisation of pain and pain-related symptoms in people with dementia. A bidirectional relationship between pain and neuropsychiatric symptoms is probable. Pain can be a major underlying cause of neuropsychiatric symptoms [7, 26] and neuropsychiatric symptoms such as depression can amplify pain related-sensitivity and distress [27]. Further, treatment for pain seems to have an effect on neuropsychiatric symptoms, and vice versa [9]. Much of the literature for this is in people with advanced dementia in hospital or living in long-term care, where neuropsychiatric symptoms that accompany pain are aggression and anxiety [26] and depression, anxiety, agitation and aggression [10, 28] in these respective settings. Less understood is the neuropsychiatric profile of people living in their own homes in the community, who may have less advanced dementia. Various neuropsychiatric symptoms including depression and agitation have been associated with pain in community-dwelling people with dementia but these associations are inconsistent [29, 30]. Our positive finding of disinhibition in this population is potentially interesting in that it may be an early, more subtle pain-associated symptom in milder dementia, compared with agitation and aggression in more advanced dementia. A lack of apathy could mean that in this population, people are more open to interventions and there is a greater chance that different management approaches are helpful.

Given the association between pain and neuropsychiatric symptoms in dementia, clinical management should be based on simultaneous, proactive assessment of both pain and neuropsychiatric symptoms, and intervening in either to influence both. Interventions that are effective for both pain and dementia are typically complex, multimodal interventions with a mixture of non-pharmacological and pharmacological approaches, targeting the person with dementia, their caregiver and the environment [9]. For example, the Serial Trial Intervention developed a toolkit for stepwise interventions incorporating structured pain assessment and comfort care before introducing pain medications [31]. Neurobiologically, it is not known whether interventions aimed at altering neural pain processing pathways have a role in alleviating these symptoms, and pain, or both. Pharmacological interventions such as gabapentinoids or specific antidepressants such as duloxetine may improve pain and specific neuropsychiatric symptoms (e.g. anxiety) but may also worsen cognition [32, 33] and are relatively contraindicated in people with dementia. Non-pharmacological interventions aimed at improving both pain and neuropsychiatric symptoms are varied [34] but evidence on their effectiveness is limited. Other experimental interventions such as brain stimulation have limited evidence for efficacy in people with cognitive impairment, although mixed evidence exists for people with traumatic brain injury [35].

Understanding pain and associated neuropsychiatric symptoms may also help with clinical management. Firstly, it may help in considering options of interventions, including combined approaches that may improve both pain and neuropsychiatric symptoms. For example, analgesic medications aimed at improving pain could reduce certain neuropsychiatric behaviours such as depression and agitation [9]. Non-pharmacological interventions such as distraction techniques, keeping to a routine, focusing on calm environments aimed at reducing disinhibited behaviours, could also result in improved pain. Secondly, it may help to define potential indicators of treatment success and pragmatic outcomes for pain and associated symptoms, where the aim of treatment is not to cure but to manage troublesome symptoms to improve quality of life. Thirdly, it may help to identify behaviours or symptoms that would complicate the management of pain intended to be managed with analgesic drugs. In these cases, careful monitoring of not just pain measurements but of neuropsychiatric symptoms themselves would be warranted – and highlighting their close association would help clinicians to be mindful of this.

### Strengths and limitations

This study researched an understudied population (community-dwelling older people with dementia) with much potential for future pain management intervention. A key strength of our findings was the descriptive characterisation of the neuropsychiatric symptom profile in the presence of pain in community-dwelling people living with mild to moderate dementia.

Dyadic study design has multiple proposed advantages for people with dementia, allowing for a supportive environment with someone who knows the person with dementia well, and helping to overcome the ethical aspects of conducting research in vulnerable people [36]. In addition, multiple perspectives, both subjective and objective, may increase reliability and accuracy of responses, than if people with dementia were interviewed alone. Methodologically, this was also reinforced in the practical undertaking of the study, in which dyad partners both facilitated and supported participation in the study for the person with dementia, and contributed towards the observation and reporting of symptoms. A disadvantage of recruiting in dyads is of limiting recruitment to people with a caregiver or family member who is available and willing to participate, potentially.

introducing selection bias, and that the dyad partner may subconsciously misrepresent responses of the person with dementia. Dyad partners’ familiarity with the person with dementia could also be variable depending on the amount of time spent with them, and could potentially introduce reporting bias.

A further limitation was that the three pain scales (NRS, BPI-SF and EQ5D3L) had differing timeframes for recall of pain severity. This was due, in part, to specific and fixed wording of the scales used. This may have introduced recall bias for pain responses. However our finding that pain reported by the same person agreed over three different scales, notwithstanding these timeframe differences, was valuable and gave insight into the person’s perception and memory of pain over time. The cross-sectional nature of this study also limited any inferences with regards to causality.

Another limitation of this study was its small sample size. Recruitment of people with dementia to clinical studies has always been challenging [37] and made more so for this study where pain is under-recognised and often dismissed even by the person living with dementia as being a common problem with ageing [38]. Notwithstanding this known and anticipated difficulty, this study did not recruit to target (*n* = 100). As a result, other genuine relationships between neuropsychiatric symptoms and pain may not have reached statistical significance. This is likely given that most neuropsychiatric symptoms were more common in people with pain even though this did not reach statistical significance. Although the significant results may have been observed by chance, there is value in identifying other pain-related symptoms in further studies with larger samples. However, this is likely to be challenging given the low rate of recruitment despite sustained efforts through multiple avenues (community engagement, memory clinics, GP surgeries, etc.). Large cohort study datasets of people with dementia may provide an alternative area for data analysis to enable these associations to be explored further.

## Conclusion

Community-dwelling people living with dementia and pain can report pain consistently using different self-reported pain scales. Certain neuropsychiatric symptoms (e.g. disinhibition) may be more common in the presence of pain but requires further study with larger cohorts. The presence of pain and associated neuropsychiatric symptoms may appear different depending on the stage of dementia.

## Data Availability

The data that support the findings of this study are available from the authors upon reasonable request and with permission of the University of Nottingham. Enquiries should be sent to jemima.collins@nottingham.ac.uk.
